# From Recognition to Rights: Suicidal Risk and Protective Factors Among Trans and Gender Diverse Youth in Japan

**DOI:** 10.1080/26895269.2025.2544901

**Published:** 2025-08-14

**Authors:** Adam O. Hill, Noriyo Kaneko, Stuart Gilmour, Lily Miyata, Ruby Grant, Teddy Cook, Adam Bourne, Natalie Amos

**Affiliations:** a School of Medicine, Nagoya City University, Nagoya, Japan; b Australian Research Centre in Sex, Health and Society, La Trobe University, Melbourne, Australia; c St. Luke’s Graduate School of Public Health, St Luke’s International University, Tokyo, Japan; d Institute of Human Rights Studies, Kansai University, Osaka, Japan; e Kirby Institute, UNSW Sydney, Sydney, Australia

**Keywords:** Suicidal ideation, Suicide attempt, Transgender youth, Harassment, Homelessness, Japan

## Abstract

**Background:**

Trans and gender diverse youth experience disproportionately high rates of suicidal ideation and suicide attempts globally. In Japan, which has one of the highest youth suicide rates among OECD countries, research on this population remains limited.

**Aims:**

This study aimed to identify demographic and psychosocial factors associated with suicidal ideation and suicide attempts in the past 12 months among trans and gender diverse youth in Japan.

**Methods:**

Data were drawn from a 2024 national cross-sectional online survey. Analyses focused on 1,693 trans and gender diverse participants aged 15–25 years. Multivariable logistic regressions were conducted to identify correlates of past-year suicidal ideation and suicide attempts.

**Results:**

In total, 45.2% of participants reported suicidal ideation and 14.7% reported a suicide attempt in the past year. Harassment, social exclusion based on gender identity, and recent homelessness were strongly associated with both suicidal ideation and attempt in the past 12 months. In contrast, disclosure of gender identity to friends was associated with reduced odds of suicidal ideation. Suicide attempts were less common among participants aged 21–25 and more common among those not currently in education.

**Conclusions:**

Findings underscore the urgent need for targeted suicide prevention strategies for trans and gender diverse youth in Japan. National policy must address systemic drivers such as harassment, exclusion, and homelessness, while expanding access to gender-affirming peer and community supports.

## Introduction

Many trans people live happy and healthy lives. However, adolescence can be an especially challenging period for trans young people, during which time they may be subject to microaggressions, discrimination, and violence (Hill et al., 2021). Accordingly, trans youth face disproportionately high rates of suicidal ideation, attempts, and suicide deaths globally, with studies from North America, Europe, and Australia consistently documenting significantly elevated risk compared to their cisgender peers (Hill et al., 2021, 2022; Patel, 2022; Reisner et al., 2016).

Despite this well-established disparity, research on suicide among trans youth in East Asia remains limited, particularly in Japan (Sakai & Tanifuji, 2021). This gap is concerning given that Japan has one of the highest youth suicide rates in the world, ranking third globally as of 2022 (OECD, 2016, 2024), and youth suicides are increasing even as adult rates decline (Dhungel et al., 2019). While the Japanese government has implemented national suicide prevention strategies, none specifically address the needs of trans youth (Ministry of Health, Labor and Welfare, Japan, 2024). Without targeted interventions, this population remains largely overlooked in public health and mental health policies (Ministry of Health, Labor and Welfare, Japan, 2024).

Preliminary data suggest that LGBT youth in Japan experience suicidal ideation and attempts at rates three to four times higher than their cisgender, heterosexual peers (ReBit, 2022). However, these studies do not disaggregate data between trans and cisgender lesbian, gay, or bisexual youth, obscuring the unique experiences and vulnerabilities of trans people (ReBit, 2022). Additionally, Japan lacks explicit legal protections against discrimination based on sexual orientation or gender identity (Human Rights Watch, 2023). Although trans people are increasingly visible in Japanese media, representations are often sensationalized or comedic, failing to translate into tangible legal rights. Recent high-profile suicides, such as that of Ryuchell, a 27-year-old Japanese media personality and LGBTQ+ advocate, underscore the ongoing challenges trans people face in navigating a society where they may be celebrated as public figures yet denied fundamental legal rights and protections (“Ryuchell tragedy shows danger of abuse against sexual minorities,” 2023).

Beyond social stigma, Japan**’**s legal and structural barriers further compound trans youth**’**s vulnerabilities. Until 2023, trans people seeking legal gender recognition were required to undergo sterilization, a policy widely condemned as a human rights violation (*Japan’s transgender law revisions should be grounded in autonomy | Human Rights Watch, 2024*, 2024). While this requirement has been repealed (although it is important to note that it has yet to be amended), Japan**’**s *Gender Identity Disorder Special Cases Act* still mandates psychiatric evaluation, being unmarried, having no children under 18, and undergoing surgery to align one**’**s physical appearance with their affirmed gender. (*Japan****’****s transgender law revisions should be grounded in autonomy | Human Rights Watch*, 2024). These restrictive policies place significant burdens on trans people, contributing to economic precarity, marginalization, and barriers to accessing gender-affirming care, all of which are linked to heightened suicide risk (*Amnesty International*, 2023). Additionally, while revisions to the *Guidelines on the Diagnosis and Treatment of Gender Identity Disorder* in 2012 permitted hormone therapy from age 15 and secondary sexual characteristic suppression therapy from Tanner Stage 2, access remains highly regulated, requiring prolonged clinical observation, out-of-pocket payment, and mandatory reporting to the Japanese Society of Psychiatry and Neurology**’**s Committee on Gender Identity Disorder for individuals under 18 (Japanese Society of Psychiatry and Neurology, Committee on Gender Identity Disorder, 2012). Together, these legal and medical constraints create significant obstacles to timely, affirming care for trans youth in Japan.

International research has identified key risk factors for suicidal ideation and suicide attempts among trans youth, including transphobic discrimination, verbal, physical, or sexual harassment or assault, and social exclusion (Callander et al., 2018; Haas et al., 2011; Hill et al., 2023; Lytle et al., 2016). Socioeconomic challenges such as housing instability and homelessness, lower educational attainment, and limited employment opportunities further increase vulnerability (Haas et al., 2011). While protective factors remain underexplored (Riggs et al., 2024), studies suggest that gender euphoria (the positive psychological impact of feeling affirmed in one**’**s gender), family and community support, and affirming healthcare experiences can mitigate suicide risk (Grant, Amos, Cook, et al., 2024; Grant, Amos, McNair, et al., 2024; Hill et al., 2023; McGowan et al., 2024; Moody & Smith, 2013) However, much of this literature originates from North American, European and Australian contexts, where cultural norms, healthcare systems, and family structures differ significantly from Japan. Culturally specific influences such as the role of family religiosity, patterns of community connectedness, and the challenges of navigating gender affirmation in Japan**’**s healthcare system remain unexamined.

This study seeks to fill these critical knowledge gaps by presenting the one of the largest studies to date examining suicidal ideation and attempts specifically among trans youth in Japan. Drawing on data from a 2024 national online survey of LGBTQA+ youth, including 1,693 trans youth aged 15–25, this study examines demographic, social, and psychosocial factors associated with recent suicidal ideation and suicide attempt, as well as potential protective factors such as community connection and disclosure of sexuality or gender identity to friends. By identifying which trans subpopulations are at elevated risk and what factors mitigate or exacerbate suicidal ideation and attempts, this research provides essential evidence for policymakers, healthcare providers, educators, and community organizations. Findings from this study can inform culturally responsive suicide prevention strategies and improve support systems for trans youth in Japan, addressing a long-standing gap in both research and policy.

Based on international research, we hypothesised that experiences of harassment, exclusion and homelessness would be associated with increased odds of suicidal ideation and suicide attempts among trans and gender diverse youth in Japan in the past 12 months. Conversely, we anticipated that educational attainment, age, and disclosure of gender identity to peers and family would be associated with lower levels of suicidal ideation and suicide attempt.

## Methods

Data for this analysis were drawn from the *Writing Themselves In Japan* (WTIJ) survey, a cross-sectional, anonymous online survey conducted between May and July 2024. The WTIJ survey examined the health and wellbeing of lesbian, gay, bisexual, pansexual, transgender or gender diverse, queer, and asexual (LGBTQA+) young people aged 15–25 years residing in Japan. Eligibility criteria included self-identifying as LGBTQA+, being between 15 and 25 years old, and currently living in Japan. Participation was voluntary and no monetary compensation was offered.

Most participants were recruited through targeted paid social media advertisements across platforms popular with young people in Japan (e.g., Instagram, TikTok). These advertisements featured an animated promotional video and/or illustrated characters designed by a multicultural LGBTQA+ creative professional. The visuals and accompanying text were collaboratively developed and refined in consultation with Japanese LGBTQA+ youth to ensure cultural relevance and community resonance. Additional recruitment was conducted through partnerships with Japanese LGBTQA+ organisations and community networks, which promoted the survey on their websites and social media platforms. Printed promotional cards featuring scannable QR codes linking to the survey were also distributed at select LGBTQA+ events and community spaces. Participants were not offered any monetary compensation for completing the survey.

Before starting the survey, participants were presented with a detailed study description and asked to confirm their eligibility. In accordance with guidelines set by the St. Luke’s International University Human Research Ethics Committee, parental consent was waived for participants under 18 years old. Participants were asked to report their age, with options ranging from *“*13 years old*”* to *“*26 years or older.*”* Those outside the eligible range (13, 14, or 26+) were automatically screened out and blocked from re-entering using browser cookies. Participants who selected age 15 were asked to confirm their current educational status. Those enrolled in junior high school were excluded, in accordance with the ethical requirements of St. Luke’s InternationalUniversity Ethics Committee, which stipulated that participants must have completed compulsory education or be enrolled in secondary school (typically ages 15–18 in Japan). In Japan, junior high school is part of the compulsory education system and distinct from secondary school. Accordingly, 35 junior high school students aged 15 were excluded. We acknowledge this may limit generalisability to younger adolescents, who may also be at heightened risk for suicidal ideation and suicide attempts. (Patel, 2022). For this paper, we focus exclusively on trans participants (*n* = 1,693). Trans participants were those whose gender identity or expression differed from the sex presumed at birth, including trans men, trans women, and non-binary individuals. Participants who did not answer questions on suicidal ideation or suicide attempts, or who selected “prefer not to answer” for these questions, were excluded from the final analytic sample. Ethics approval for the WTIJ study was granted by the St. Luke’s International University Human Research Ethics Committee.

## Measures

The WTIJ survey was adapted from the Australian Writing Themselves In 4 (WTI4) study (Hill et al., 2021) through an iterative process involving LGBTQA+ experts, community advisors, and youth participants in both Australia and Japan. Development of the original WTI4 instrument included extensive consultation with a community advisory group made up of LGBTQA+ experts, including researchers and representatives from advocacy organisations, with specific input from a gender advisory panel comprised of trans professionals and one academic. This community-engaged model was carried forward in Japan, where all survey items and translations were reviewed by a local LGBTQA+ advisory members that included researchers, community members with lived experience, and trans and gender diverse youth. Feedback was collected online and in person and informed several key changes to ensure cultural and linguistic appropriateness. Advisory members across both settings were compensated for their time and contributions unless participation was part of a salaried role or declined by the individual.

This study was led by a multidisciplinary team including cisgender and trans researchers, with guidance from LGBTQA+ youth advisors and community experts. Reflexive discussions throughout the research process, including during data interpretation, helped ensure that the analytic lens remained attentive to the lived realities of trans and gender diverse youth in Japan. Our approach aimed to centre community-informed knowledge while navigating structural limitations related to gender categorisation in quantitative research.

### Demographics

Participants provided their age, sexual orientation (gay, lesbian, bisexual, pansexual, heterosexual/straight, asexual, unsure/questioning, and *“*something different*”*), ethnic background (Japanese or other/mixed), educational setting (no schooling, secondary school, university, 2-year university/vocational school, or other), engagement in paid employment (yes/no), and residential location (capital city inner/outer suburban, regional city or town, rural/remote).

Regarding gender, participants were first asked to indicate their sex assumed at birth and then to answer, *“*Which options best describe your gender?*”* Respondents could select multiple responses from five options: *“*male,*” “*female,*” “*non-binary,*” “*I use a different term,*”* and *“*gender questioning/unsure.*”* Those who selected any option other than *“*male*”* or *“*female,*”* chose more than one option, or whose reported gender did not align with their sex assumed at birth were subsequently asked, *“*Which of the following additional options best describes your gender?*”* This follow-up question presented 17 gender identity options, developed based on existing literature, the foundational Australian study (Hill et al., 2021), and refined through consultations with Japanese trans and gender diverse community members. Respondents were permitted to select multiple options and, if they did, were invited to indicate the term they felt most closely represented their gender in a subsequent question, or to indicate that they did not feel comfortable choosing a single term.

Although this multi-stage approach captured a broad range of self-reported identities, including culturally specific gender terms such as x-jendā, chūsei, ryōsei, and musei, the number of participants selecting these terms was very small. This limitation reflects both sampling realities and broader structural constraints: gender diversity in Japan remains poorly institutionalised, and gender categories outside of the male-female binary are often fragmented, locally situated, or unrecognised in formal systems. Japanese gender categories emerge from cultural and historical contexts distinct from Western LGBTQA+ identity labels, and are often not self-identifying (Dale, 2012; Robertson, 1998; Takeuchi, 2020) complicating efforts to directly compare or aggregate gender identities in large-scale survey research. As a result, and in close consultation with trans and gender diverse experts and community partners, we combined all non-cisgender identities into a single *“*trans and gender diverse*”* category for analytic purposes. This decision ensured statistical validity in our regression analyses while aligning with best practice for preserving self-identification and inclusion (Puckett et al., 2020). While this limits the possibility of disaggregated gender identity analysis, it allowed us to analyse correlates of suicidal ideation and suicide attempt among trans and gender diverse youth in Japan a statistically robust and interpretable way. A planned follow-up study, led by trans researchers in Japan, will explore the broader gender diversity captured in this survey in depth, including culturally specific identities.

### Suicidal ideation and suicide attempts

Suicidal ideation and attempts in the past 12 months were assessed using two questions: one on thoughts of suicide, wanting to die, or ending one**’**s life, and another on any suicide attempts. Response options were *“*no,*” “*yes, in the past 12 months,*” “*yes, more than 12 months ago,*”* and *“*prefer not to answer.*”* For this study, we examined only recent (past 12 months) ideation and attempts. Prior to showing these questions, we provided information regarding helplines and a content warning noting that *“*skipping these questions does not make your answers any less valuable.*”* Participants who indicated discomfort with the subject matter of these questions were allowed to skip them; those who skipped or answered “prefer not to answer” for suicidal outcomes were excluded from the respective analyses.

### Disclosure and familial religiosity

Disclosure of sexuality or gender identity to family and friends was assessed with the item, *“*Have you come out to or talked with any friends or family about your sexuality or gender identity?*”* Participants responded separately for family and friends, selecting from the options *“*none of them,*” “*a few of them,*” “*some of them,*” “*most of them,*” “*all of them,*”* or *“*not applicable.*”* Responses were categorized as *“*none,*” “*a few/some,*” “*most/all,*”* or *“*not applicable*”* for analytical purposes.

Family or household religiosity was assessed using a yes/no question, *“*Is your family or household religious?.*”*

### Harassment and exclusion

Experiences of harassment or assault based on sexuality, gender identity, and/or gender expression in the past 12 months were assessed using a series of items. Participants were asked whether they had experienced verbal, physical, or sexual harassment or assault specifically due to their sexuality, gender identity, or gender expression. Each form of harassment or assault was listed separately with examples to aid understanding. For physical harassment or assault, examples included being shoved, punched, or injured with a weapon. For sexual harassment or assault, examples included unwanted touching, sexual remarks, sexual messages, or being forced to perform an unwanted sexual act. Participants could select any experiences that applied or indicate that they had not experienced any of the listed forms. For the purposes of analysis in this paper, responses were combined into a single binary variable indicating whether participants had experienced any form of sexuality- or gender-based harassment or assault (yes/no). Feelings of deliberate exclusion based on sexuality or gender identity in the past 12 months were also recorded (yes/no).

### Homelessness

Homelessness was examined in the *Writing Themselves In Japan* survey using questions adapted from a large-scale US study of 26,161 young people (Morton et al., 2018) and the WTI4 survey conducted in Australia (Hill et al., 2021). Participants were asked whether they had experienced running away, being asked to leave, couch surfing, or homelessness - either within the past 12 months or at any time in their lives. Internationally recognized frameworks from the Australian Bureau of Statistics (ABS) (Australian Bureau of Statistics, 2018) and the Organisation for Economic Co-operation and Development (OECD) (OECD, 2023) informed our definition. The ABS defines homelessness as inadequate living arrangements lacking security of tenure, autonomy, or sufficient space for social relationships (Australian Bureau of Statistics, 2018), while the OECD characterizes it as lacking access to a safe, secure, and affordable home that ensures privacy, safety, and opportunities for social connection (OECD, 2023). In contrast, Japan**’**s definition focuses on “visible homelessness” - individuals residing in public spaces such as parks, riverbanks, streets, or train stations (OECD, 2023). To address these differences, our study adopted the broader international concept of homelessness, ensuring consistency with global research and enhancing the applicability of our findings. For the purposes of the present study analyses, a dichotomous variable indicating whether or not participants had experienced recent (past 12 months) homelessness was generated.

### LGBT social connections

Social connection was assessed through two items that asked participants about their participation in LGBT groups or events over the past 12 months. The question, "In the past 12 months, how often have you participated in an LGBT group or event?" was presented separately for in-person and online contexts. Response options for each included: “Never,” “Once,” “Monthly,” and “Weekly.” For the purposes of analysis in this study, responses to both items were recoded into a single binary variable indicating any participation (online or in-person) versus no participation.

## Statistical analyses

Analyses were conducted using Stata (Version 16.1 SE, StataCorp, College Station, TX, USA). Descriptive statistics described sample characteristics. Univariable logistic regressions were performed to identify variables associated with suicidal ideation and attempts. Variables with *p*-values <0.250 in univariable analyses were considered for inclusion in the multivariable models, ensuring potentially relevant factors were not omitted prematurely (Bursac et al., 2008; Hosmer et al., 2013). Two multivariable logistic regression models were fitted: one for suicidal ideation and another for suicide attempts. Variance inflation factors (VIFs) were computed to check for multicollinearity. All VIFs were below 2.0, indicating no serious collinearity concerns. Adjusted odds ratios (AORs), 95% confidence intervals (CIs), and *p*-values were reported for both models.

## Results

### Sample characteristics

Table 1 displays descriptive statistics for all study variables. The majority of participants were aged 18–25 years, with fewer (14.6%) aged 15–17 years. Regarding sexual orientation, the most common identity was pansexual (19.5%), followed by something else (17.4%), bisexual (16.0%), and unsure (15.0%). Smaller proportions identified as lesbian (11.0%), asexual (8.9%), gay (7.1%), and heterosexual (5.2%).

**Table 1.** Descriptive statistics for all study variables (*N* = 1,598).

	Number (*n*)	%
Age group (years)		
15–17	233	14.6
18–20	726	45.4
21–25	639	40.0
Sexual orientation		
Lesbian	176	11.0
Gay	113	7.1
Bisexual	255	16.0
Pansexual	312	19.5
Heterosexual	83	5.2
Asexual	142	8.9
Unsure	239	15.0
Something else	278	17.4
Ethnic background		
Japanese	1,513	94.8
Other/mixed	83	5.2
Current educational setting		
No schooling	518	32.4
secondary school (high school)	272	17.0
university	628	39.3
2-Year university/vocational school	106	6.6
Other	73	4.6
Currently engaged in employment		
No	431	27.1
Yes	1,162	72.9
Family or household Religious		
No	1,313	82.3
Yes	282	17.7
Residential location		
Capital city, inner suburban	134	8.4
Capital city, outer suburban	326	20.5
Regional city or town	614	38.7
Rural/Remote	513	32.3
Disclosed sexuality or gender identity to family		
No	631	39.5
A few/some	486	30.4
Most/all	215	13.4
Not applicable	267	16.7
Disclosed sexuality or gender identity to friends		
No	235	14.7
A few/some	1,031	64.7
Most/all	238	14.9
Not applicable	90	5.6
Any verbal, physical or sexual harassment in past 12 months based on sexuality or gender identity		
No	1,198	75.6
Yes	387	24.4
Felt deliberately left out or excluded in past 12 months based on sexuality or gender identity		
No	1,407	88.7
Yes	179	11.3
Experienced any homelessness in past 12 months		
No	1,499	94.2
Yes	93	5.8
In the past 12 months have you participated in an LGBT group or event in person?		
No	1,317	82.7
Yes	275	17.3
In the past 12 months have you participated in an LGBT group or event online?		
No	1,464	93.4
Yes	103	6.6
Suicidal ideation in past 12 months		
No	864	54.8
Yes	714	45.2
Suicide attempt in past 12 months		
No	1,281	85.3
Yes	220	14.7

**Table 2.** Regression results for suicidal ideation in past 12 months (*N* = 1,496)

	Suicidal ideation		Univariable regression		Multivariable regression	
Variable	Number (*n*)	(%)	OR (95% CI)	*p*-Value	AOR (95% CI)	*p*-Value
Age group (years)						
15–17	133	57.3	REF		REF	
18–20	318	44.5	0.60 (0.44–0.80)	0.001	0.67 (0.40–1.13)	0.131
21–25	263	41.7	0.53 (0.39–0.72)	0.000	0.64 (0.36–1.13)	0.121
Sexual orientation						
Gay	53	46.9	REF		REF	
Lesbian	80	46.0	0.96 (0.60–1.55)	0.878	1.16 (0.69–1.96)	0.582
Bisexual	123	48.8	1.08 (0.69–1.68)	0.736	1.33 (0.81–2.18)	0.268
Pansexual	157	50.8	1.17 (0.76–1.80)	0.478	1.42 (0.87–2.30)	0.158
Heterosexual	25	30.1	0.49 (0.27–0.89)	0.019	0.49 (0.25–0.96)	0.038
Asexual	55	39.0	0.72 (0.44–1.20)	0.207	0.86 (0.49–1.49)	0.589
Unsure	97	42.0	0.82 (0.52–1.29)	0.389	1.00 (0.60–1.65)	0.998
Something else	124	45.3	0.94 (0.60–1.45)	0.768	1.06 (0.65–1.74)	0.803
Ethnic background						
Japanese	669	44.8	REF		REF	
Other/mixed	43	53.1	1.40 (0.89–2.18)	0.145	1.35 (0.80–2.27)	0.261
Current educational setting						
secondary school (high school)	147	54.6	REF		REF	
No schooling	222	43.4	0.64 (0.47–0.86)	0.003	1.05 (0.61–1.80)	0.873
University	253	40.9	0.58 (0.43–0.77)	0.000	0.90 (0.54–1.49)	0.672
2-Year university/vocational school	51	48.1	0.77 (0.49–1.21)	0.254	1.12 (0.59–2.10)	0.734
Other	40	55.6	1.04 (0.61–1.75)	0.891	1.20 (0.62–2.34)	0.594
Currently engaged in employment						
No	209	48.7	REF		REF	
Yes	502	43.9	0.82 (0.66–1.03)	0.089	0.99 (0.74–1.31)	0.922
Family or household Religious						
No	578	44.7	REF		REF	
Yes	135	48.2	1.15 (0.89–1.49)	0.280		
Residential location						
Capital city, inner suburban	68	50.7	REF		REF	
Capital city, outer suburban	134	41.2	0.68 (0.45–1.02)	0.063	0.66 (0.42–1.02)	0.061
Regional city or town	250	41.7	0.70 (0.48–1.01)	0.058	0.64 (0.42–0.96)	0.030
Rural/Remote	259	50.9	1.01 (0.69–1.47)	0.977	0.96 (0.64–1.46)	0.860
Disclosed sexuality or gender identity to family						
None	284	45.4	REF		REF	
A few/some	227	47.2	1.08 (0.85–1.37)	0.546	1.12 (0.86–1.46)	0.406
Most/all	85	40.3	0.81 (0.59–1.12)	0.199	1.10 (0.74–1.63)	0.638
Not applicable	118	45.4	1.00 (0.75–1.34)	0.996	1.06 (0.74–1.50)	0.759
Disclosed sexuality or gender identity to friends						
None	112	48.1	REF		REF	
A few/some	473	46.5	0.94 (0.71–1.25)	0.658	0.79 (0.57–1.09)	0.150
most/all	90	38.0	0.66 (0.46–0.96)	0.027	0.47 (0.30–0.75)	0.001
Not applicable	37	43.5	0.83 (0.51–1.37)	0.473	0.75 (0.41–1.37)	0.347
Any verbal, physical or sexual harassment in past 12 months based on sexuality or gender identity						
No	481	40.8	REF		REF	
Yes	226	58.5	2.05 (1.62–2.59)	0.000	1.92 (1.47–2.51)	0.000
Felt deliberately left out or excluded in past 12 months based on sexuality or gender identity						
No	596	43.0	REF		REF	
Yes	111	62.4	2.20 (1.59–3.03)	0.000	1.63 (1.12–2.36)	0.010
Experienced any homelessness in past 12 months						
No	634	42.9	REF		REF	
Yes	77	82.8	6.41 (3.71–11.10)	0.000	6.97 (3.93–12.35)	0.000
In the past 12 months have you participated in an LGBT group or event in person?						
No	586	45.1	REF			
Yes	123	45.4	1.01 (0.78–1.32)	0.926		
In the past 12 months have you participated in an LGBT group or event online?						
No	658	45.5			REF	
Yes	39	39.0	0.77 (0.51–1.16)	0.207	0.64 (0.41–1.03)	0.064

**Table 3.** Regression results for suicide attempt in past 12 months (*N* = 1,461).

	Suicide attempt		Univariable regression		Multivariable regression	
Variable	Number (*n*)	(%)	OR (95% CI)	*p*-Value	AOR (95% CI)	*p*-Value
Age group (years)						
15–17	45	20.8	REF		REF	
18–20	102	15.0	0.67 (0.45–0.99)	0.044	0.70 (0.37–1.30)	0.255
21–25	73	12.1	0.52 (0.35–0.79)	0.002	0.36 (0.19–0.71)	0.003
Sexual orientation						
Gay	18	16.7	REF		REF	
Lesbian	27	16.9	1.02 (0.53–1.95)	0.964	1.30 (0.64–2.62)	0.471
Bisexual	38	16.5	0.98 (0.53–1.82)	0.960	1.16 (0.58–2.30)	0.677
Pansexual	44	14.8	0.87 (0.48–1.58)	0.638	1.16 (0.60–2.24)	0.651
Heterosexual	7	9.1	0.50 (0.20–1.26)	0.143	0.51 (0.18–1.45)	0.205
Asexual	20	14.7	0.86 (0.43–1.73)	0.675	1.08 (0.51–2.31)	0.833
Unsure	27	12.2	0.69 (0.36–1.32)	0.265	1.06 (0.53–2.15)	0.864
Something else	39	14.6	0.85 (0.46–1.57)	0.605	1.10 (0.56–2.15)	0.784
Ethnic background						
Japanese	210	14.8	REF			
Other/mixed	9	11.5	0.75 (0.37–1.53)	0.431		
Current educational setting						
secondary school (high school)	46	18.1	REF		REF	
No schooling	83	16.9	0.92 (0.62–1.37)	0.689	2.10 (1.10–3.99)	0.024
University	59	10.0	0.50 (0.33–0.76)	0.001	0.84 (0.45–1.57)	0.583
2-Year university/vocational school	17	17.5	0.96 (0.52–1.77)	0.899	1.45 (0.65–3.20)	0.363
Other	15	21.7	1.26 (0.65–2.42)	0.495	1.74 (0.82–3.67)	0.147
Currently engaged in employment						
No	64	16.0	REF			
Yes	153	14.0	0.86 (0.62–1.17)	0.334		
Family or household Religious						
No	179	14.5	REF			
Yes	40	15.0	1.04 (0.72–1.51)	0.835		
Residential location						
Capital city, inner suburban	20	15.9	REF		REF	
Capital city, outer suburban	34	11.0	0.66 (0.36–1.19)	0.165	0.70 (0.37–1.33)	0.278
Regional city or town	85	14.9	0.93 (0.54–1.57)	0.774	0.98 (0.55–1.75)	0.945
Rural/Remote	81	16.7	1.07 (0.62–1.82)	0.817	1.17 (0.65–2.08)	0.602
Disclosed sexuality or gender identity to family						
None	87	14.7	REF		REF	
A few/some	75	16.2	1.12 (0.80–1.57)	0.507	0.95 (0.65–1.37)	0.768
Most/all	30	14.6	0.99 (0.63–1.54)	0.949	0.87 (0.54–1.40)	0.559
Not applicable	28	11.5	0.75 (0.48–1.19)	0.222	0.61 (0.37–1.01)	0.055
Disclosed sexuality or gender identity to friends						
None	31	14.2	REF			
A few/some	138	14.2	1.01 (0.66–1.53)	0.974		
Most/all	37	16.4	1.19 (0.71–1.99)	0.516		
Not applicable	13	15.9	1.14 (0.57–2.31)	0.711		
Any verbal, physical or sexual harassment in past 12 months based on sexuality or gender identity						
No	121	10.8	REF		REF	
Yes	98	26.5	2.97 (2.21–4.01)	0.000	2.71 (1.94–3.80)	0.000
Felt deliberately left out or excluded in past 12 months based on sexuality or gender identity						
No	174	13.2	REF		REF	
Yes	43	25.3	2.23 (1.52–3.26)	0.000	1.47 (0.96–2.26)	0.077
Experienced any homelessness in past 12 months						
No	178	12.6	REF		REF	
Yes	41	47.7	6.30 (4.01–9.89)	0.000	6.02 (3.77–9.62)	0.000
In the past 12 months have you participated in an LGBT group or event in person?						
No	184	14.9	REF			
Yes	34	13.1	0.86 (0.58–1.28)	0.466		
In the past 12 months have you participated in an LGBT group or event online?						
No	198	14.4	REF			
Yes	14	15.1	1.06 (0.59–1.90)	0.853		

Most participants were of Japanese ethnic background (94.8%). Regarding educational background, two-fifths (39.3%) attended university and 17.0% secondary school.

In terms of religious affiliation, 17.7% of participants reported having a religious family or household. The majority of participants resided in regional cities or towns (38.7%) or rural or remote areas (32.3%), with 28.9% in capital cities.

### Suicidal ideation and attempts

With respect to experiences of suicidal ideation and attempts, 45.2% of participants reported experiencing suicidal ideation, and 14.7% suicide attempt in the past 12 months.

### Disclosure

The majority (85.3%) had disclosed to any friends, while 60.5% had disclosed to any family members.

### Harassment, exclusion, and homelessness

A total of 24.4% of participants reported experiencing verbal, physical, or sexual harassment based on their sexuality or gender identity in the past 12 months. Additionally, 11.3% of participants reported feeling deliberately left out or excluded, and 5.8% reported experiencing homelessness in the past year.

### Participation in LGBT groups or events

In the past 12 months, 17.3% of participants reported participating in an LGBT group or event in person, while 6.6% reported participating in such groups or events online.

## Regression analyses

### Suicidal ideation in the past 12 months

Table 2 displays the multivariable model for suicidal ideation, several significant associations were identified. Heterosexual participants were significantly less likely to experience suicidal ideation compared to gay participants (AOR = 0.49, 95% CI: 0.25–0.96, *p* = 0.038). Participants residing in regional cities or towns had lower odds of suicidal ideation compared to those in inner suburban capital cities (AOR = 0.64, 95% CI: 0.42–0.96, *p* = 0.030). Additionally, participants who disclosed their sexuality or gender identity to most or all friends had significantly lower odds of suicidal ideation compared to those who had not disclosed to any friends (AOR = 0.47, 95% CI: 0.30–0.75, *p* = 0.001).

Participants who experienced verbal, physical, or sexual harassment based on their sexuality or gender identity had significantly increased odds of suicidal ideation (AOR = 1.92, 95% CI: 1.47–2.51, *p* < 0.001). Similarly, participants who reported feeling deliberately left out or excluded in the past 12 months were more likely to report suicidal ideation (AOR = 1.63, 95% CI: 1.12–2.36, *p* = 0.010). Experiencing homelessness in the past 12 months was strongly associated with suicidal ideation, with an almost sevenfold increase in odds (AOR = 6.97, 95% CI: 3.93–12.35, *p* < 0.001).

### Suicide attempts in the past 12 months

Table 3 displays the multivariable model for suicide attempts, significant findings included that participants aged 21–25 years had significantly lower odds of attempting suicide compared to those aged 15–17 years (AOR = 0.36, 95% CI: 0.19–0.71, *p* = 0.003). Participants not currently in education had higher odds of attempting suicide compared to those in secondary school (AOR = 2.10, 95% CI: 1.10–3.99, *p* = 0.024).

Participants who experienced verbal, physical, or sexual harassment based on their sexuality or gender identity had increased odds of attempting suicide (AOR = 2.71, 95% CI: 1.94–3.80, *p* < 0.001). Additionally, participants who had experienced homelessness in the past 12 months had markedly higher odds of attempting suicide (AOR = 6.02, 95% CI: 3.77–9.62, *p* < 0.001).

## Discussion

This study is the first large-scale study that focuses specifically on suicidal ideation and attempts among trans youth in Japan. Our findings reinforce global evidence showing disproportionately high rates of suicidal ideation and suicide attempts in trans populations (Hill et al., 2021, 2022; Patel, 2022; Reisner et al., 2016), but they also highlight critical nuances in the Japanese context. Although trans and gender diverse individuals are relatively visible in Japanese media (Mackie, 2008), our data make it clear that this surface-level visibility does not guarantee day-to-day safety or mental well-being for trans youth. Elevated rates of suicidal ideation (45.2%) and attempts (14.7%) in our sample were comparable to those observed in North American research and the foundational Australian study (Grossman & D**’**Augelli, 2007; Hill et al., 2021; Kosciw et al., 2020), yet markedly higher than rates observed among the general youth population in Japan. For example, a 2022 online survey conducted by the Nippon Foundation involving 18,320 youth from the general population found that 6.4% had experienced suicidal ideation and 2.1% had attempted suicide in the past year (The Nippon Foundation, 2022), rates that are approximately seven times lower than those observed in this study, demonstrating that heightened visibility alone may be insufficient to address the underlying social stressors and systemic barriers that trans youth confront.

Several risk and protective factors were identified through the analyses. First, experiences of sexuality and gender-based harassment and deliberate exclusion were strongly associated with higher odds of both suicidal ideation and attempts, echoing international literature (Goldblum et al., 2012; Haas et al., 2011). Second, homelessness, defined in accordance with broader global frameworks (OECD, 2023), had a profound effect on suicidal ideation, with nearly a sevenfold increase in odds for trans youth experiencing housing instability. This contrasts starkly with Japan**’**s narrower focus on *“*visible homelessness*”* (e.g., sleeping rough in public spaces), suggesting that many vulnerable trans youth, including those couch surfing or displaced from family homes, may go unrecognized by official statistics (OECD, 2023). Although the present study could not directly assess pathways into homelessness, international evidence suggests that experiences of family rejection, abuse, and exclusion, particularly related to gender identity, are among the most common drivers of homelessness among trans youth (40,41). It is likely that the mistreatment identified in our study, including harassment and social exclusion, may also contribute to housing instability for some trans young people in Japan. These findings point to a pressing need for trans affirming youth housing programs that explicitly include and protect trans people who lack stable or affirming living arrangements.

Furthermore, our findings indicate that younger trans youth aged 15–17 years had significantly higher odds of suicide attempts compared to their older peers aged 21–25 years. This aligns with international research observing that younger trans youth are at significantly elevated risk of suicidal ideation and attempts compared to their older peers, particularly when they lack family and peer support (Patel, 2022; Reisner et al., 2016), but it contrasts with the mortality profile of youth suicide in Japan among the general population, where university-aged young people had much higher mortality rates than high school or junior high school-aged children (Dhungel et al., 2019). This difference in profile between attempts and mortality may reflect the higher pressures trans youth face compared to their peers of the same age. Similarly, participants residing in regional cities and towns reported lower odds of suicidal ideation compared to those in inner suburban capital cities. While previous research has highlighted urban areas as sites of increased LGBTQA+ visibility and support, some studies have also suggested that regional areas, despite having fewer LGBTQA+ resources, may provide greater community cohesion and social connection, factors that can mitigate mental health risks (Bain, 2022; Grant et al., 2023; Ryan et al., 2010; A. J. Smith et al., 2018). Moreover, qualitative research suggests that some trans youth migrate from rural to urban areas seeking acceptance, yet face isolation, poverty, and difficulty accessing services upon relocation, exacerbating mental health distress (Patel, 2022).

Disclosure of gender identity also played a significant role in mental health outcomes. Being out to most or all friends was protective against suicidal ideation, highlighting the importance of peer support in mitigating the effects of transphobia and stigma. However, disclosure to family appeared more complex, with a substantial proportion of participants having not disclosed their gender identity to any family members. This may reflect cultural norms around preserving family harmony, intergenerational gaps in understanding gender diversity, or fear of rejection (Charbonnier & Graziani, 2016; Motoyama, 2019; Tamagawa, 2017). It also suggests that while peer networks can provide critical support, many trans youth lack comparable acceptance at home, where rejection or lack of understanding may further compound mental health vulnerabilities, and/or increase risk of homelessness.

Despite some gains in public acceptance of gender diversity, such as greater media visibility, our findings suggest that trans youth in Japan remain vulnerable to systemic discrimination and mental health stressors. While some trans people have achieved prominence in Japanese entertainment and media, this visibility does not necessarily translate into broader societal acceptance. Public opinion data indicate that Japan is among the least supportive countries regarding increased protections for transgender people, with only 41% of respondents agreeing that their country should do more to support trans rights and 43% of Japanese respondents believed their country was becoming more tolerant of transgender people - the second lowest among countries surveyed (Clark & Jackson, 2018). This disconnect between trans visibility in public life and the lack of meaningful protections has real consequences.

The death of Ryuchell in 2023 brought national attention to the social pressures faced by individuals who do not conform to normative gender roles in Japan. Known widely as a media personality and advocate, Ryuchell experienced intense public scrutiny following the announcement of their divorce, during which it was disclosed that they no longer identified with the role of a heterosexual husband. Ryuchell**’**s death underscored the profound challenges at the intersection of visibility, gender role expectations, and mental health in contemporary Japan (Radford & Oi, 2023; “Ryuchell tragedy shows danger of abuse against sexual minorities,” 2023). The tension between public recognition and systemic discrimination suggests that trans people in Japan may face unique stressors, including being accepted as entertainers but denied fundamental rights in legal, medical, and workplace settings. In Japan, emphasis on fitting in and adhering to societal norms may lead to heightened feelings of isolation and marginalization for those who deviate from expected gender roles. Compared to Western contexts, where individualism and personal identity may be prioritized (Kazama & Kawaguchi, 2010; McLelland, 2000), the communal nature of Japanese society means that exclusion can result in deeper psychological distress and barriers to support. However, communal societies may also offer opportunities for promoting inclusion, particularly when norms shift in affirming directions. Previous research in Japan suggest that interventions which emphasise peer-led support initiatives and inclusive community networks may be effective in addressing stigma and promoting belonging (Kaneko et al., 2020).

In response, policymakers must strengthen anti-discrimination laws to address harassment in education and public life, while also broadening the official definition of homelessness to recognize less visible forms of housing instability and improving social welfare support for homeless young people, who are particularly vulnerable to suicide. Mental health services need to prioritize culturally attuned, gender-affirming care that recognizes the role of extended kin networks and social harmony in shaping how trans youth disclose and navigate their identities. Training school nurses, healthcare and education professionals to identify early signs of distress, offer trauma-informed support, and foster inclusive environments is equally crucial. Lastly, interventions led by young people themselves, particularly peer-based community groups, may be especially effective in reaching individuals reluctant to engage with formal services or worried about negative repercussions of seeking help.

### Limitations

This study represents the largest sample of trans and gender diverse youth in Japan to date, providing critical findings regarding their mental health. However, this study has several limitations. First, the data were collected via an online survey, which may limit the generalizability of findings to all trans youth in Japan. While online recruitment is often the most effective approach for reaching marginalized populations, it may exclude trans youth who lack internet access or those who are hesitant to participate in LGBTQA+-focused research due to privacy concerns.

Second, this study employed a convenience sampling method, a widely used approach in suicide research for hard-to-reach populations, such as LGBTQA+ individuals and people experiencing homelessness (Balakrishnan et al., 2022; Hill et al., 2020; B. C. Smith et al., 2016). While this method is effective in capturing the experiences of trans youth, it does not allow for reliable prevalence estimates of suicidal ideation and suicide attempts in in this sample. However, previous studies using similar sampling strategies have consistently found high rates of suicidal ideation and suicide attempts among trans youth in both Japan and other countries, including the Australian foundational study this is based on (Hill et al., 2022; ReBit, 2022). Moreover, due to survey promotion through social media and community-based recruitment, young people in rural or remote areas may be underrepresented, as algorithmic ad targeting and limited connection to LGBTQA+ networks may reduce reach in these regions.

A further limitation is our focus on recent (past 12 months) suicidal ideation and suicide attempt. Lifetime suicidality captures cumulative risk factors and long-term exposure which differ substantially from proximal predictors (e.g., harassment, housing instability), as empirical studies show distinct patterns of association and timing. Moreover, leading LGBTQA+ youth health studies treat these outcomes separately to preserve temporal specificity and inform targeted policy responses (e.g., Hill et al., 2023; Kaplan et al., 2016). Future studies should examine the correlates of lifetime suicidal ideation and suicide attempts among trans youth to better capture long-term and acute predictors.

A key limitation of this study is the necessity of collapsing gender identity responses into a single *“*trans and gender diverse*”* category. While the survey instrument was designed to capture a wide range of identities, including culturally specific terms relevant to Japan, small subgroup sizes made disaggregated analysis statistically unfeasible. Our decision to combine all non-cisgender respondents into a single category was made in consultation with trans and gender diverse advisors and aligns with established methodological recommendations for research involving small or emergent identity groups (Puckett et al., 2020). While this analytic choice protected the integrity and interpretability of the results, it limits intra-group comparisons, among which there may be important differences with regards to suicidal ideation and suicide attempt have been observed (Rimes et al., 2019). This research should be studied qualitatively in future research to provide a better understanding of these potential differences. Importantly, a separate, trans community-led study is currently in development to further analyse the culturally specific gender identities reported by participants in this survey in greater depth.

Lastly, self-reported data on suicidal ideation and attempts may be subject to social desirability or recall bias (Kelly et al., 2013). However, given that this study was conducted anonymously online, the likelihood of social desirability bias is lower than in face-to-face interviews or clinical settings. Despite these limitations, this study offers an important foundation for future research. In particular, community-led and co-produced qualitative research is essential to deepen understanding of culturally specific identities, lived experiences, and the structural barriers shaping mental health among trans youth in Japan.

## Conclusion

Our findings reveal critically high rates of suicidal ideation and attempts among trans and gender diverse youth in Japan, challenging the perception that a rise in public visibility may translate into a diminished risk of self-harm. Widespread experiences of harassment, exclusion, and hidden homelessness, coupled with restrictive or unsupportive family environments, drive these mental health outcomes. At the same time, the protective role of being out to supportive peers underscores the value of affirming social networks. Taken together, these insights call for policy reforms, expanded definitions of homelessness and associated interventions, trans affirming mental health care, and stronger legal protections against discrimination. Ultimately, bridging the gap between superficial acceptance and true inclusivity will require collective efforts across government agencies, healthcare providers, educators, and youth-led community initiatives committed to ensuring that trans young people in Japan not only gain visibility but also experience meaningful safety, acceptance, and care.
